# Multitargeted Molecular Docking and Dynamic Simulation Studies of Bioactive Compounds from *Rosmarinus officinalis* against Alzheimer’s Disease

**DOI:** 10.3390/molecules27217241

**Published:** 2022-10-25

**Authors:** Fatima Javed Mirza, Saadia Zahid, Sanila Amber, Sumera, Hira Jabeen, Noreen Asim, Syed Adnan Ali Shah

**Affiliations:** 1 Neurobiology Laboratory, Atta ur Rahman School of Applied Biosciences, National University of Sciences and Technology, Islamabad 44000, Pakistan; 2 Center for Computational Chemistry, School of Chemistry, University of Bristol, Bristol BS8 1TH, UK; 3 Institute of Biotechnology and Genetic Engineering, The University of Agriculture, Peshawar 25130, Pakistan; 4 Faculty of Pharmacy, Universiti Teknologi MARA Cawangan Selangor Kampus Puncak Alam, Bandar Puncak Alam 42300, Malaysia; 5 Atta-ur-Rahman Institute for Natural Products Discovery (AuRIns), Universiti Teknologi MARA Cawangan Selangor Kampus Puncak Alam, Bandar Puncak Alam 42300, Malaysia

**Keywords:** Alzheimer’s disease, *Rosmarinus officinalis*, AChE, BACE I, Synapsin I, II, III, docking

## Abstract

Alzheimer’s disease (AD) has been associated with the hallmark features of cholinergic dysfunction, amyloid beta (Aβ) aggregation and impaired synaptic transmission, which makes the associated proteins, such as β-site amyloid precursor protein cleaving enzyme 1 (BACE I), acetylcholine esterase (AChE) and synapsin I, II and III, major targets for therapeutic intervention. The present study investigated the therapeutic potential of three major phytochemicals of *Rosmarinus officinalis,* ursolic acid (UA), rosmarinic acid (RA) and carnosic acid (CA), based on their binding affinity with AD-associated proteins. Detailed docking studies were conducted using AutoDock vina followed by molecular dynamic (MD) simulations using Amber 20. The docking analysis of the selected molecules showed the binding energies of their interaction with the target proteins, while MD simulations comprising root mean square deviation (RMSD), root mean square fluctuation (RMSF) and molecular mechanics/generalized born surface area (MM/GBSA) binding free energy calculations were carried out to check the stability of bound complexes. The drug likeness and the pharmacokinetic properties of the selected molecules were also checked through the Lipinski filter and ADMETSAR analysis. All these bioactive compounds demonstrated strong binding affinity with AChE, BACE1 and synapsin I, II and III. The results showed UA and RA to be potential inhibitors of AChE and BACE1, exhibiting binding energies comparable to those of donepezil, used as a positive control. The drug likeness and pharmacokinetic properties of these compounds also demonstrated drug-like characteristics, indicating the need for further in vitro and in vivo investigations to ascertain their therapeutic potential for AD.

## 1. Introduction

AD is a debilitating disorder characterized by the progressive loss of cognition, learning and memory. It is the most common age-related neurodegenerative disorder which can lead to the loss of bodily functions and death [1]. The major neuropathological hallmarks of AD include the amyloid beta aggregates, which occur extracellularly, and the abnormally phosphorylated interneuronal fibrillar tau proteins [2]. Although there is a high prevalence of sporadic AD, several genetic factors govern the progression of the disease, particularly mutations in amyloid precursor protein (APP), presenilin 1 (PSEN1) and presenilin 2 (PSEN2), resulting in familial forms of AD (fAD) [1]. In addition, dysfunctional signaling of acetylcholine (ACh), deteriorated synaptic transmission and increased production of Aβ by β-site amyloid precursor protein cleaving enzyme 1 (BACE1) are integral in mediating AD progression [3,4].

In recent years, plant and other natural product-derived lead compounds have garnered significant attention. These natural products, including herbs and spices, possess various phytochemicals which serve as potential sources of natural antioxidants and neuroprotectants and are devoid of the potentially life-threatening side effects characteristic of the existing approved drugs [5,6]. The currently available drugs for the symptomatic treatment of AD, such as donepezil, tacrine, rivastigmine, and galantamine, demonstrate observable adverse reactions such aa hepatotoxicity and gastrointestinal effects including vomiting, diarrhea, and nausea, reducing their suitability to be used by the patients [7,8].

*Rosmarinus officinalis* (*R. officinalis)* from the family *Lamiaceae* is a woody perennial herb, indigenous to the Mediterranean region, and has been widely used as a condiment in addition to its application for diverse medicinal purposes, being particularly recognized as a stimulant, mild analgesic, choleretic, anticancer, and hepatoprotective agent [9,10]. *R. officinalis* has gained sufficient attention among the herbs and the spices as a rich source of phytochemicals such as carnosic acid (CA), rosmarinic acid (RA), ursolic acid (UA), and camphor, which demonstrate antioxidant, anti-inflammatory, and anticarcinogenic properties [11,12,13]. It also exhibits analgesic, anti-anxiety and memory -nhancing effects, necessitating further research on its active constituents for the development of therapeutic agents against nervous system disorders like AD, Parkinson’s disease and epilepsy [14].

RA, a phenolic ester, is abundantly present in the herbs belonging to the family *Labiatae*. It possesses various biological and pharmacological activities, including anti-oxidant, anti-mutagenic, and anti-apoptotic activities [15]. RA also plays a beneficial role against AD through the suppression of Aβ aggregation [16]. Additionally, it has been found to be effective against copper (II)-induced neurotoxicity through the formation of an original ternary association between amyloid β and Cu (II) [17]. A recent study also highlights the prevention of fibrillization and the assembly of β sheets in tau protein, thereby suggesting its therapeutic potential against AD [18].

CA, another polyphenolic diterpene derived from rosemary, is known to reverse the Aβ_25–35_-induced loss of cell viability in human neuroblastoma SH-SY5Y cells [19]. It also plays a protective role in cognitive impairment due to Aβ-induced neurotoxicity observed in animal models [20,21]. Furthermore, CA was also found to play a role in the attenuation of the risk of ApoE4-associated AD [22].

**Figure 1:**
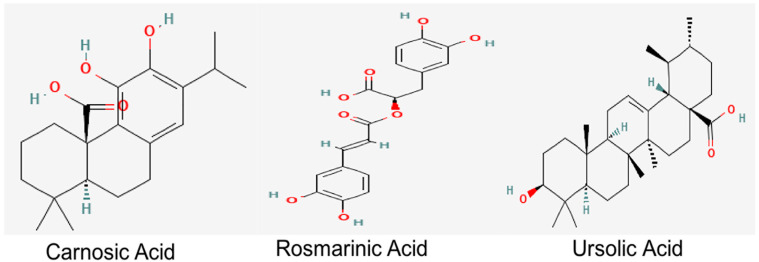
Chemical structures of bioactive constituents of *R. officinalis.* Structures were acquired from PubChem database.

UA, a natural pentacyclic triterpenoid, which is also present in abundant levels in *R. officinalis*, provides health benefits against oxidative stress, inflammation and fibrosis [23,24]. Derivatives of UA also exert cholinesterase-inhibiting potential [25]. Studies conducted on an Aβ-induced mouse model also demonstrated the role of UA in the prevention of cognitive impairment through the amelioration of oxidative stress and inflammation [26]. RA and UA have also been reported to alleviate the cognitive deficits, synaptic dysregulation and the associated neurodegeneration in an Aβ-induced AD model, thereby reiterating their therapeutic significance against AD [27]. Their structures are depicted in Figure 1.

The computer-aided in silico approach has been widely employed for the initial stages of drug discovery. The prediction analysis of the best possible drug candidates for various diseases through the in silico approach [28,29,30] is not only efficient and cost-effective but also reduces errors in the final steps. The present study was designed to identify the potential drug targets of the three major constituents of *R. officinalis*, i.e., RA, CA and UA. These compounds have shown neuroprotective effects in a previous study by our group [27], therefore, to get further insight on their potential protein targets, the current study was planned and their binding potentials with acetylcholine esterase (AChE), β-site amyloid precursor protein cleaving enzyme 1 (BACE1), and synapsin I, II and III were assessed. In AD, AChE causes the breakdown of acetylcholine in the synapses, resulting in disrupted cholinergic transmission. It also interacts directly with amyloid beta to increase its deposition into insoluble plaques [31].

BACE1 (beta-site amyloid precursor protein cleaving enzyme 1) is another prime AD target protein as it is involved in the generation of amyloid beta, which aggregates into plaques and thereby contributing to AD pathology. BACE1 inhibition in early stages of AD can help in slowing the production of amyloid beta [32]. Similarly, synaptic loss has been implicated in the cognitive alterations associated with AD. Restoration of the levels of synaptic proteins such as synapsin I, II and III can serve as a treatment strategy against AD, thereby making them target proteins [33].

Donepezil has been used as a positive control as it is a second-generation cholinesterase inhibitor approved by the FDA for the treatment of mild, moderate and severe AD and is widely prescribed to control the dementia associated with AD. It acts by the reversible inhibition of the enzyme acetylcholinesterase thereby reducing the associated neurodegeneration and synaptic loss [34,35]. Various studies have also shown significant effects of donepezil on BACE1 expression. A study by Sarno et al. reported significant reduction and downregulation in the protein expression of BACE1 in patients treated with chronically with donepezil. This effect may be interpreted as evidence of disease modification [36]. It is also evident that various analogues of donepezil also exhibit inhibitory activity against BACE1 [37]. Donepezil was thereby used for a comparative assessment of the binding potential of RA, UA and CA against protein hallmarks of AD.

Molecular docking simulations are among the most widely accepted methods for computer-aided drug designing as they predict receptor–ligand interactions at the molecular level, thereby helping to identify potential drug candidates in a comparatively short period of time. These tools provide assistance for the wet lab experiments by generating a list of promising candidates on which experiments can be performed in an informed fashion, hence reducing the overall cost of drug discovery [38,39].

Moreover, a reliable molecular docking analysis depends on the accuracy of the adopted scoring function that is used to determine the binding mode and site of a ligand, predict binding affinity and identify the potential drug leads for a given protein target [40]. Perhaps an imprecision in the binding site of the target protein and selection of an inappropriate docking pose and inconsistency with MD simulations are the most frequently encountered issues associated with docking studies [41]. Numerous software tools, based on different algorithms and physicochemical approximations, have been developed for molecular docking in recent years. Among these, AutoDock Vina is one of the most widely cited, open-source applications reported to exhibit the best docking power among all the docking methods tested in a comparative assessment of scoring functions (CASF) 2013 [42]. It also exhibited the best scoring power as assessed by its estimation of binding affinity among ten common docking programs [43]. However, it has certain shortcomings and has been found to struggle with the correct identification and scoring of the crystal structures of ligands in benchmark studies [44]. In addition, it does not support modeling specific features such as macrocycles or explicit water molecules. Recent amendments have implemented this functional deficiency in AutoDock Vina 1.2.0 and combined the scoring function of AutoDock 4.2, along with the concurrent docking of multiple ligands and a batch mode for docking a sizeable count of ligands [45]. Integration of a scoring function correction term improves the protein–ligand docking and screening accuracies that substantially facilitate the prediction abilities for the docking of AutoDock Vina and screening tasks based on CASF-2016, DUD-E and DUD-AD [46]. Amendments in certain empirical parameters may also improve the ligand ranking of AutoDock Vina [47]. Similarly, hardware acceleration can minimize the irregular computations and reduce the execution runtimes of AutoDock [48].

For a better efficiency of computer aided drug designing, MD simulations coupled with docking studies could improve the binding mode prediction and scoring of the protein–ligand complexes and ultimately aid the discovery of lead compounds. Thereby the current study applied computer aided molecular docking analysis and simulation coupled with the prediction of drug likeness and pharmacokinetic properties to assist in elucidating the therapeutic potential of these compounds against AD.

## 2. Results

### 2.1. Molecular Docking Studies of Rosmarinic, Carnosic and Ursolic Acid on BACE1, AChE, Synapsin I, II and III

Molecular docking studies were used to estimate the receptor–ligand interaction geometrics for the selected compounds. The docking scores for RA, CA, UA and donepezil with interacting BACE1, AChE and synapsin I, II and III residues, including hydrogen bonds and van der Waals interacting residues, are stated in Table 1. The potential of RA, CA and UA against the AD target molecules was linked with the binding energy of the interactions and the associated hydrogen bonding (Table 1).

#### 2.1.1. RA and UA Exhibit Binding Energy Comparable to Donepezil in Binding Interactions with AChE

**Figure 2:**
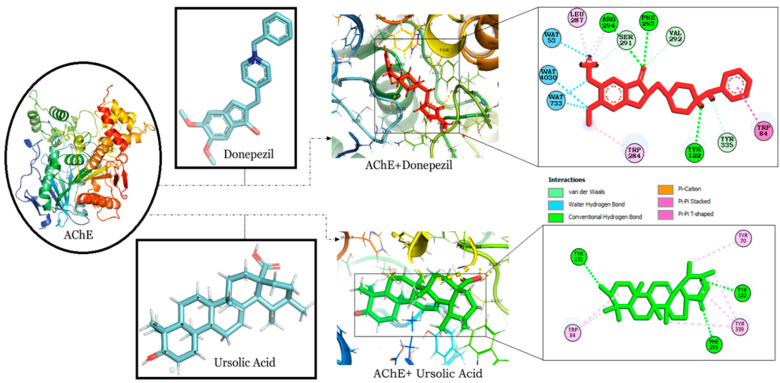
Molecular docking interaction models of AChE (PDB ID: 4PQE) with donepezil and ursolic acid. 2D structures of the compounds are shown by line and stick models with the surrounding amino acids of AChE. The interactions are denoted by the following colors: hydrogen bonding interactions (green), alkyl bonds (pink) and bumps (red). AChE; Acetylcholinesterase.

The binding interaction of RA, CA and UA with AChE revealed that amongst the other compounds, RA, with a binding energy of −9.56, had a better affinity than CA (binding energy: −7.91) and UA (binding energy: −9.17). However, the binding energy of UA was the same as that of donepezil and it was found to bind to the same binding pocket as that of donepezil, forming hydrogen and alkyl bonds with similar residues and suggesting the binding and inhibition of AChE (Table 1). Interestingly, both UA and donepezil formed hydrogen bonds with Tyr 122 and Phe 293 (Figure 2).

#### 2.1.2. RA Exhibits Strong Binding Interactions Strikingly Similar to Donepezil with BACE1

**Figure 3:**
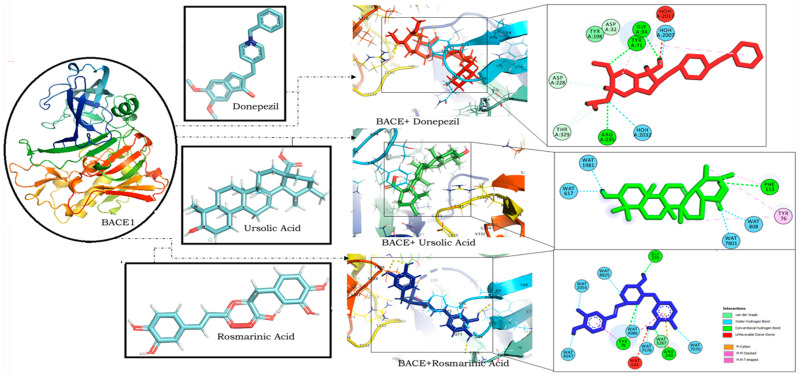
Molecular docking interaction models of BACE 1 (PDB ID: 2WJO) with donepezil, ursolic acid and rosmarinic acid. 2D structures of the compounds are shown by line and stick models with the surrounding amino acids of BACE1. The interactions are denoted by the following colors: hydrogen-bonding interactions (green), alkyl bonds (purple) and bumps (red). BACE1; β-site amyloid precursor protein cleaving enzyme 1.

RA was found to have the lowest binding energy in comparison to CA (Binding energy: −5.85) and UA (binding energy: −5.48) in interaction studies with BACE1. With a binding energy of −7.45, it exhibited a strong binding potential comparable to that of donepezil (binding energy: −8.27) (Table 1). It also bound to similar residues and at the same binding site as that of donepezil (Figure 3).

#### 2.1.3. RA Exhibits Strong Binding Interactions with Synapsin I, II and III

Docking studies of RA, CA and UA with synapsin I also demonstrated a stronger binding affinity of RA with synapsin I in comparison to UA, CA and donepezil. The binding energy of the interaction of RA with synapsin I was −8.49 which was even lower than that of donepezil (binding energy: −6.5) (Table 1).

Similarly, RA had a binding energy of −7.02 in its binding with synapsin II, which was lower than that of UA (binding energy: −6.02) and CA (−5.08) and comparable to that of donepezil (binding energy: −6.50). This suggests a stronger binding potential of RA to synapsin II.

**Figure 4:**
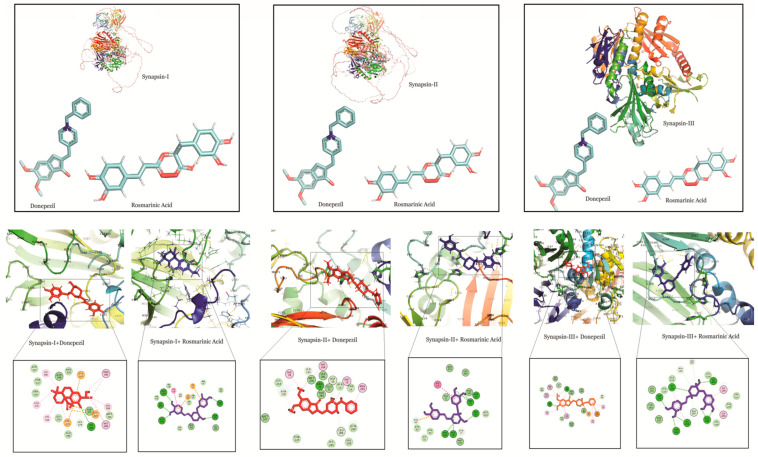
Molecular docking interaction models of synapsin I, II and III with donepezil and rosmarinic acid. 2D structures of the compounds are shown by line and stick models with the surrounding amino acids of synapsin I, II and III. The interactions are denoted by the following colors: hydrogen bonding interactions (green), carbon bonds (blue), alkyl bonds (purple) and bumps (red).

In interactions with synapsin III, RA demonstrated a higher binding potential with a binding energy of −8.05. UA, CA and donepezil exhibited higher values, showing their comparatively lower binding potential (Table 1). A study of the interacting residues further revealed that RA exhibited binding with synapsin I, II and III through the same binding pocket as that of donepezil (Figure 4).

### 2.2. Molecular Dynamic Simulation Studies of Rosmarinic, Carnosic and Ursolic Acid on BACE1, AChE, Synapsin I, II and III

An MD simulation was conducted to evaluate the flexibility and overall stability of docked complexes. RMSD and RMSF graphs were generated to determine the residual deviations and fluctuations in the complexes. Figures reveal the residual deviation and fluctuations of docked complexes. An increasing trend was observed in all the complexes, having diverse RMSD values at the equilibrium state (starting) of 0 to 25 ns in the simulation period.

**Figure 5:**
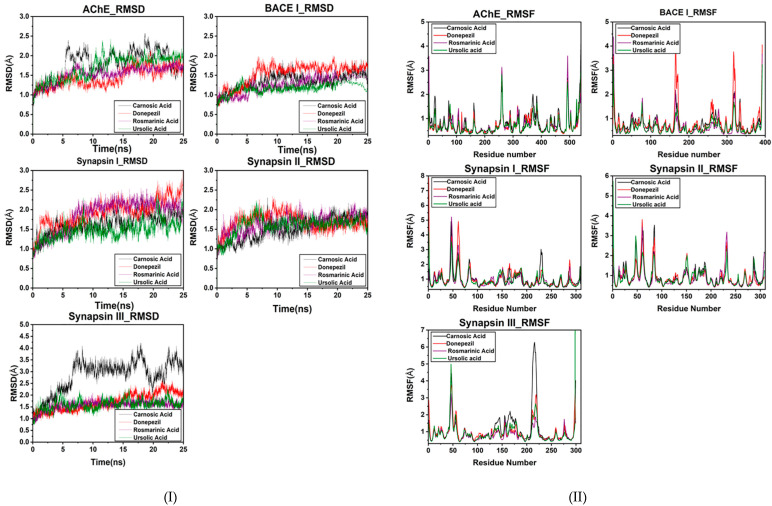
(**I**) RMSD plot of the bioactive compounds of *R. officinalis* with target proteins at 25 ns. (**II**) RMSF plot of AChE, BACE1, synapsin I, synapsin II and synapsin III with CA, RA, UA and donepezil respectively.

In the docked poses of compounds with AChE, RA, CA and UA demonstrated stable complexes throughout the simulation time in comparison to that of donepezil. Stability trends can be observed in the RMSD plots. The RMSD values of the docked poses with BACE I also had values of less than two, showing good reproducibility of the docked pose. The RMSD profile of synapsin I and II also exhibits a good binding orientation, as the values are less than three. CA exhibited a less stable binding with synapsin III than RA and UA, which showed a similar RMSD profile to that of donepezil (Figure 5I).

The overall MD results also showed the fluctuations during the simulation time. The graphs suggested that most of the complexes had little fluctuation throughout the simulation period. The RMSF results of compounds with AChE reveal that UA had fluctuations comparable to those of donepezil throughout the simulation period. CA exhibited comparatively higher fluctuations, depicting a less stable bond.

The BACE1 RMSF profile with the compounds showed that UA had the highest binding stability with BACE1. Comparative analyses revealed that RA also exhibited fluctuating peaks similar to those of CA, while donepezil was observed to have the highest number of fluctuated peaks signifying its poor stability. RA also exhibited the least fluctuated peaks in the RMSF profile with synapsin I and II and III (Figure 5II). The stable behaviors of docked complexes throughout the MD trajectories validate the docking results, thereby increasing their efficacy.

### 2.3. Binding Free Energies of Interactions

**Figure 6:**
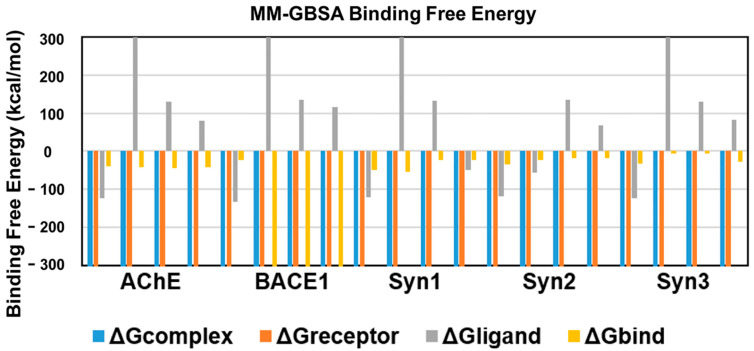
Binding free energies of bioactive compounds of *R. officinalis* against AChE, BACE1, synapsin I, synapsin II, and synapsin III with CA, RA, UA and donepezil, respectively.

An energetic analysis was conducted to acquire the binding affinities using the MM/GBSA method with the protein–ligand complexes. The scores obtained from the MM/GBSA calculations are shown in Table 2. The overall binding free energies of the complexes of AChE with all the ligands was ~40 kcal/mol, expect for ursolic acid with ${\Delta G}_{\mathrm{bind}}$ = −43.7367 kcal/mol, which revealed the most stable complex following the measurement of the docking affinities trend, as shown in Figure 6.

BACE1 exhibited the lowest binding free energy $({\Delta G}_{\mathrm{bind}}$) values and showed the most stable complexes against all ligands. UA with a ${\Delta G}_{\mathrm{bind}}$ value of −2610.6 kcal/mol demonstrated the most stable bond in comparison to RA (−23.6095), CA (−2541.98) and donepezil (−2538.656), in consistence with the results of RMSD and RMSF.

The binding free energy of CA with synapsin I was −54.3 kcal/mol, which is lower than that of RA, UA and donepezil and depicts the stability of the complex. However, RA showed favorable binding energies with that of synapsin II and III. With a binding energy of −35.1 kcal/mol with synapsin II and −32.49 kcal/mol with synapsin III, it exhibited more stable complexes than those of donepezil, UA and CA (Table 2, Figure 6).

### 2.4. Drug Likeness Analysis of CA, RA and UA

The prediction of the drug likeness of the compounds was carried out through a Lipinski filter, ADMETSAR and SwissADME to analyze their drug-like characteristics and assess their pharmacokinetic properties. All of the three compounds exhibited properties indicative of their potential to be used as therapeutic agents. Their molecular masses were less than 500 Daltons and they displayed less than five hydrogen bond donors and less than ten acceptors. The molar refractivity as well their cLogP values were also in accordance with the Lipinski rule. The compounds demonstrated comparable results on all other parameters of the Lipinski filter, ADMETSAR and SwissADME, thereby suggesting their drug likeness and suitability to be suggested as therapeutic agents against AD (Table 3).

## 3. Discussion

The current study was performed to elucidate the therapeutic potential of three major constituents of the polar nature of *R. officinalis*, i.e., RA (polyphenol), CA (labdane-type diterpene) and (UA) pentacyclic triterpenoid. Therefore, molecular docking was performed with these compounds to reveal their binding affinity and interaction with the target proteins of AD in comparison to donepezil.

We used AutoDock Vina for the current analysis, which applies an automated protocol for the prediction of receptor–ligand binding and has thereby been widely applied in computer-aided drug designing [49]. For typical systems, AutoDock is run several times to give several docked conformations, and am analysis of the predicted energy and the consistency of results is combined to identify the best solution. AutoDock Vina generates more accurate binding poses, while a better binding affinity is formed in AutoDock4; perhaps both the programs are highly successful for a huge data set of diverse protein–ligand complexes [50]. We chose best the pose of the protein–ligand-complexes on the basis of the highest scoring from top 10 poses of the ligand binding sites.

All the selected ligand molecules (CA, RA, UA) were docked successfully against their targets (AChE, BACE1, synapsin I, II, III). The ligand molecules that had the lowest binding energy or docking score were considered as the best ligand molecules in inhibiting the target receptor, as a lower binding energy corresponds to higher binding affinity.

AD pathology has been attributed to cholinergic dysfunction according to the ‘cholinergic hypothesis’, which believes cholinergic deficit to be the major culprit for short-term memory deficits [51]. In our study, we have targeted AChE as an important AD-associated protein, as it hydrolyzes acetylcholine (ACh) and causes the termination of cholinergic signaling. Its inhibition has been widely studied and most of the drugs for AD, such as donepezil, galantamine and rivastigmine, are inhibitors of AChE [52]. Among the other drugs, donepezil is a highly selective and reversible inhibitor of AChE, which is effective in improving cognitive and behavioral deficits in AD patients [53]. The interaction of donepezil with AChE occurs along the active-site gorge of the enzyme involving the catalytic site, the acyl pocket and the peripheral anionic binding site, through the adoption of outward–inward–inward orientations. The interaction comprises of reversible axial displacement and the reorientation of donepezil at the active site, mediated by water molecules. [54]. This study reveals the high affinity of RA and UA towards AChE, as depicted by their binding energies of −9.56 and −9.17 respectively (Table 1). MD simulation also optimized the interactions to predict complex flexibility and to investigate the stability of the complex, which revealed a stable confirmation as observed in RMSD and RMSF plots. MM-GBSA binding free energy values also support the docking studies, as UA exhibited the lowest binding free energy value of −43.73 in comparison to RA, CA and donepezil. Additionally, UA and donepezil were also observed to interact with AChE through three amino acids, specifically Tyr, Phe, Trp. An analysis of the interacting residues further revealed that both the compounds formed hydrogen bonds with AChE through Tyr 122 and Phe 293 (Figure 2). The potential of UA in the attenuation of amyloid-beta-induced neurotoxicity through the regulation of the NF-κB signaling pathway is also evident [55]. It has also been reported to alleviate cognitive and synaptic deficits and restore adult hippocampal neurogenesis in an amyloid-beta-induced AD mouse model [27]. The obtained results thereby propose the significant potential of UA against AD.

Higher levels of BACE1 are also associated with AD; thereby, it is targeted as an important protein for AD mitigation [56]. A significantly higher expression of BACE1 is evident and contributes to the higher Aβ production in the AD brain in comparison to normal aging brains [32,57]. BACE1 is formed in the endoplasmic reticulum as an immature, glycosylated, pro-BACE1 propeptide, exhibiting an open and closed confirmation [58]. The ProBACE1 matures in the Golgi apparatus through the cleavage of the pro-domain, thereby resulting in activation of BACE1 [59]. This cleavage makes the catalytic active site accessible to substrate allowing BACE1 to exhibit its full enzymatic activity [60]. Activated BACE1 comprises four potential *N-*glycosylation sites and six cysteine residues which form three disulfide bonds that are essential for the activity of the enzyme [61]. It also exists in a flap-open conformation and a flap-closed conformation. The flap-open confirmation is more energetically stable, however, BACE1 adopts a flap-closed confirmation upon binding to the substrate. This shift in confirmation involves the breakage of hydrogen bonds between the oxygen of Tyr71 and the nitrogen of Gly74, the nitrogen of Lys75 and the oxygen of Glu77, and the Tyr71 hydroxyl with the Lys107 oxygen. This destabilization permits the interaction of the enzyme with its substrates. The Tyr 71 side chain interacts with the indole nitrogen of Trp 76, and the substrates can interact with the enzyme through a cleft. A bottleneck formation by Thr72, Arg235, Ser328, and Thr329 serves as a specificity mechanism requiring some flexibility in the substrate [62,63].

The interaction of RA with BACE1 has a binding energy of −7.45 (Table 1), which was further validated with the stable orientation depicted by MD simulation results. Interestingly RA was also found to interact with BACE1 through the same amino acids as that of donepezil at a similar binding site (Figure 3). RA comprises five major functional groups through which it interacts with the target proteins: a carboxy group, an unsaturated C–C bond, two phenol hydroxy groups, an alkoxy group, and an ester moiety [64]. Analysis of the interacting residues revealed that both RA and donepezil interact with BACE1 at a similar binding site, forming hydrogen bonds with similar residues. RA forms hydrogen bonds with BACE1 through Gly 235, Tyr 76 and Arg 240 while donepezil exhibits hydrogen bonds with BACE1 through Gly 34, Tyr 71 and Arg 235. The results of the MD simulation also demonstrated the stable binding orientation of RA with BACE1, as evident from the RMSD, RMSF profiles, as well as the binding free energy values. A study on RA derived from *Salvia fruticosa* reported neuroprotective effects against amyloid-beta-induced neurotoxicity through the inhibition of BACE1 [65]. These results thereby indicate the promising effect of RA in BACE1 inhibition, suggesting its potential against AD.

RA also showed the highest binding affinity for synapsin I, II and III in comparison to the other compounds (Table 1). Synaptic impairment and loss of synapses are the major consequences of AD [66]. Synapsin proteins play crucial roles in synaptic maturation and plasticity, however, the dysregulation of their expression is reported by several studies [33,67]. The involvement of synapsin I in the up-regulation of BACE1 activity and modulation of elevation of APP/BACE1 interaction that promotes the Aβ production indicates disturbed molecular mechanism(s) and the formation of aggregates in AD [68]. Interestingly, our findings demonstrate a strong affinity of RA towards both BACE1 and synapsin I. Therefore, we postulated RA as a promising agent that can suppress abnormal Aβ production by targeting BACE1 and synapsins in AD, however, further in vivo and in vitro studies on the molecular interactions are warranted. Moreover, MD simulations are more accurate and provide a flexible binding model of the receptor and ligand along with an estimation of the effect of surrounding explicit water molecules. Despite being comparatively more time consuming and incurring a higher computational cost [69,70], they are routinely incorporated as favorable approach for drug design. Likewise, the integration of structure-based virtual screening (SBVS) is robust, convenient and is one of the most promising in silico techniques for drug design [71]. Nonetheless, lead discovery based on virtual screening has been found to yield false positive results, and therefore should be followed by biological assays for a more holistic approach [72].

UA, CA and RA also satisfied the drug likeness criteria as predicted through the Lipinski filter and ADMETSAR and SwissADME analyses, which revealed their potential pharmacokinetic properties. The absorption profiles showed that all of these compounds are predicted to undergo human intestinal absorption while the permeability of UA and CA for Caco-2 also represents their passage through biological barriers [73,74]. They were also found to exhibit the promiscuous inhibition of OATP1B1 and OATP1B3 transporters, which are involved in the metabolism of drugs. Toxicity profiles also exhibited the absence of carcinogenicity and mutagenesis potential, which ultimately demonstrates the potential of the studied compounds as being comparable to already recognized medicates for AD (Table 3).

Our study showed that the active compounds UA, RA and CA of *R. officinalis* exhibit significant potential by docking with the AD target proteins AChE, BACE1 and synapsin I, II and III. These findings indicate the need for further in vitro and in vivo investigations to ascertain their therapeutic potential for the safe and effective treatment of AD.

## 4. Materials and Methods

### 4.1. Molecular Docking Simulations

In our study, RA, CA and UA were tested for their interaction with BACE1, AChE, synapsin I, II and III. The 3D structures of BACE1 (PDB ID: 2WJO), ACHE (PDB ID: 4PQE) and synapsin III (PDB ID: 2P0A) were acquired from the RCSB Protein data bank (PDB) (https://www.rcsb.org/) accessed on 15 February 2022 [75]. Synapsin I and II structures were generated through AlphaFold (https://alphafold.ebi.ac.uk/) accessed on 15 February 2022 [76]. The 3D structures of RA, CA and UA were constructed using ArgusLab (http://www.arguslab.com/arguslab.com/ArgusLab.html) accessed on 18 February 2022 [77]. AutoDOCK Vina [49] was employed to assess the structure of the receptor–ligand complex and to ascertain the feasibility of the structural topographies necessary for the interaction of the compounds derived from *R. officinalis* with AD target proteins. It allows for the exploration of possible key active site residues involved in the intermolecular interactions with the ligand. The automated docking models generated were visualized using BIOVIA Discovery Studio 2017 R2 [78]. The best pose was chosen based on the highest scoring from the top 10 poses of ligand binding sites.

### 4.2. Molecular Dynamics Simulation Analysis

All the simulations and analyses were done using Amber20 (https://ambermd.org/) accessed on 20 February 2022 [79]. Partial charges were calculated through antechamber package using semi-empirical method ‘bcc’ and missing parameters were generated through General Amber Force Field (GAFF) [80] using the LEap module. The FF14SB force field was used for proteins and GAFF was used for ligand parameterization. The structures were solvated in a 12 Å box of TIP3P water, crystal water molecules were removed following the docking protocol and existing charges were neutralized by adding sodium ions.

For MD simulation, the system was briefly minimized using 100 steepest descent and 200 conjugate gradient cycles with a restraint force of 100 kcal/mol on water, sodium, substrate, and hydrogen atoms, followed by another minimization of protein heavy atoms with a 5 kcal/mol force constant. The system was heated with 5 kcal/mol force restraints on α-carbon from 25 K to 298 K for 20ps using a canonical ensemble and then equilibrated for 1ns without restraints using NPT, followed by the production of MM MD for a total of 25 ns. All bonds involving hydrogen atoms were constrained using SHAKE to remove fast bond stretching motions and by using larger time steps (0.002 ps). To approximate longer range interactions, the particle mesh Ewald method was used with a cut off value of 8.0 Å. Trajectories were saved at every 100 picoseconds and analyzed at a stable potential energy. Free energy calculations and other physical parameters such as root mean square deviation (RMSD) and root mean square fluctuation (RMSF) were carried out to gain structural insight into the complexes. The structures were visualized using Pymol (https://pymol.org/2/) accessed on 22 February 2022 [81] and Xmgrace (http://plasma-gate.weizmann.ac.il/Grace/) accessed on 22 February 2022 [82] was used for plotting.

### 4.3. Binding Free Energy Calculation Using MM/PBSA and MM/GBSA

To study the protein–drug complex energetics and stability, binding free energy calculations are a significant tool to measure the strength of drug binding to a protein. The energetics were calculated from 50 snapshots only, due to expensive computational–time. Molecular mechanics–Poisson-Boltzmann surface area (MM-PBSA) and molecular mechanics–generalized-born surface area (MM–GBSA) are two efficient methods in AMBER to calculate binding free affinity. MM–GBSA estimates favorable binding free energies and can be calculated in the following useful way:

(1) $${\Delta G}_{\mathrm{bind}}={\;\Delta G}_{\mathrm{complex}}-{\;\Delta G}_{\mathrm{receptor}}-{\;\Delta G}_{\mathrm{ligand}}$$

(2) $${\Delta G}_{\mathrm{bind}}=\;\Delta H\;-T\Delta S\;\;\text{≈}\;{\Delta E}_{\mathrm{MM}}+{\;\Delta G}_{\mathrm{solv}}-\;T\Delta S$$

(3) $${\Delta E}_{\mathrm{MM}}={\;\Delta E}_{\mathrm{internal}}+{\;\Delta E}_{\mathrm{ele}}+{\;\Delta E}_{\mathrm{vdw}}$$

(4) $${\Delta G}_{\mathrm{solv}}={\;\Delta E}_{\mathrm{polar}-\mathrm{solvation}}+{\;\Delta E}_{\mathrm{non}-\mathrm{polar}}$$

The total binding free energy $\left({\Delta G}_{\mathrm{bind}}\right)$ is the energy difference between the complex bound state (${\Delta G}_{\mathrm{complex}})$ and the individual free receptor (${\Delta G}_{\mathrm{receptor}}$) and the ligand (${\Delta G}_{\mathrm{ligand}}$) as stated in Equation (1). The total free energy binding $\left({\Delta G}_{\mathrm{bind}}\right)$ is further decomposed, according to law of thermodynamics, into the change in enthalpy ΔH and entropy TΔS. The enthalpy change ΔH is calculated through MM–GBSA whereas entropy changes are subsumed into ${\Delta G}_{\mathrm{solv}}$, which is part of ΔH as in Equations (2)–(4) [83,84,85].

### 4.4. Prediction of Drug Likeness of CA, RA and UA

The Lipinski filter (http://www.scfbio-iitd.res.in/software/drugdesign/lipinski.jsp) was accessed on 10 March 2022, to carry out the drug likeness prediction of CA, RA and UA in accordance with the Lipinski rule of 5 [86]. This rule helps to predict the drug likeness of molecules on the basis of their compliance with two or more of the following rules: molecular mass < 500 Daltons, cLogP < 5, hydrogen bond donor < 5, hydrogen bond acceptor < 10 and molar refractivity between 40 and 130. Additionally, adsorption, distribution, metabolism, excretion and toxicity (ADMET) properties of the compounds were predicted using the ADMETSAR (http://lmmd.ecust.edu.cn/admetsar2/) accessed on 12 March 2022 and SwissADME (http://www.swissadme.ch/) accessed on 14 March 2022, which serve as important tools for the estimation of the pharmacokinetic properties of compounds to predict their drug likeness [87,88]. These tools provide assistance in determining the candidate compounds for drug discovery and development. The SDF (structure data format) files and canonical SMILES (simplified molecular-input line-entry system) of CA, RA and UA were downloaded from the PubChem database (https://pubchem.ncbi.nlm.nih.gov/) accessed on 10 March 2022 [89] to calculate the drug likeness and ADMET properties using default parameters.

## 5. Conclusions

The active compounds UA, RA and CA of *R. officinalis* showed significant potential by binding with AD target proteins AChE, BACE1 and synapsin I, II and III. Our study showed UA and RA as potent inhibitors of AChE and BACE1, while their drug likeness and pharmacokinetic properties also demonstrated their drug-like characteristics, indicating the need for further in vitro and in vivo investigations to ascertain their therapeutic potential. We believe that this study can contribute to developing new therapeutic strategies for the safe and effective treatment of AD.

## Figures and Tables

**Table 1:** 3D Docking interaction of *R. officinalis* active compounds with the target proteins.

Target	Ligand	Binding Energy	Interacting Residues
AChE	Rosmarinic acid	−9.56	H Bonds: Phe 293, Phe 336, Tyr 335, Asn 85, Glu 200 Pi-Pi interaction: Phe 295, Trp 84 Pi-Sigma: Tyr 339
AChE	Carnosic acid	−7.91	H Bonds: Ser 123, Tyr 335 Alkyl Bonds: Val 71, Pro 86 Pi-Sigma: Trp 84 Carbon-Hydrogen bond: Gly 119
AChE	Ursolic acid	−9.17	H Bonds: Tyr 131, Tyr 122, Phe 293 Pi- Alkyl Bonds: Trp 84, Tyr 70, Tyr 339
AChE	Donepezil	−9.17	H Bonds: Tyr 122, Phe 293, Arg 294 Alkyl Bonds: Leu 287, Trp 284 Pi-pi stacking: Trp 84 Carbon-hydrogen bond: Ser 291, Val 292, Tyr 335
BACE1	Rosmarinic acid	−7.45	H Bonds: Tyr 76, Gly 235, Arg 240
BACE1	Carnosic acid	−5.85	H Bonds: Asp 37, Asp 233 Alkyl Bond: Val 337, Tyr 203, Tyr 76, Phe 113 Carbon-hydrogen bond: Gly 39
BACE1	Ursolic acid	−5.48	H Bonds: Phe 113 Alkyl Bonds: Tyr 76
BACE1	Donepezil	−8.27	H Bonds: Tyr 71, Gly 34, Arg 235 Van der waals: Asp 228, Thr 329
Synapsin I	Rosmarinic acid	−8.49	H Bonds: Asp 120, Lys 403, Gln 399, Ser 275, Asn 338 Pi-anion: Asp 10, Arg 186 Alkyl Bond: His 123
Synapsin I	Carnosic acid	−7.13	H Bonds: Phe 222 Alkyl Bonds: Val 219 Pi-pi interaction: His 188
Synapsin I	Ursolic acid	−5.69	H Bonds: His 123, Ser 275 Alkyl Bonds: Ala 193, His 188, Pro 393
Synapsin I	Donepezil	−6.5	H Bonds: Lys 225 Alkyl Bonds: His 188, Val 388, Pro 393, Lys 336, Leu 375, Ile 385 Pi-Anion: Glu 373, Lys 269, Lys 279
Synapsin II	Rosmarinic acid	−7.02	H Bonds: Arg 76, Ser 281, Asp 10, His 78, His 164 Carbon-hydrogen bonds: Gln 77, Glu 86
Synapsin II	Carnosic acid	−5.08	H Bonds: Asp 120 Alkyl Bonds: Ala 124, Leu 394, His 188 Salt bridge: Arg 186 Carbon hydrogen bond: His 123, Pro 393, His 188, His 274
Synapsin II	Ursolic acid	−6.02	H Bonds: Gln 399 Carbon-hydrogen bond: Leu 394 Alkyl Bonds: Ala 124, His 123, Pro 393
Synapsin II	Donepezil	−6.50	H Bonds: Arg 76 Alkyl Bonds: Arg 293, His 78, His 164, His 13 Carbon-hydrogen Bond: Asp 10, Leu 284, Asp 228, Ser 165
Synapsin III	Rosmarinic acid	−8.05	H Bonds: Lys 106, Lys 254, Lys 150, Thr 184, Gly 152, Glu 186 Alkyl Bonds: Lys 160, Ile 266 Carbon-hydrogen bond: Ala 156, Gly 157, Glu 267,
Synapsin III	Carnosic acid	−4.59	H Bonds: Ala 316, Asp 292, Asn 317 Alkyl Bonds: Lys 315, Val 354, Phe 286, Ile 364, Val 246, Ile 287, Lys 258 Salt bridge: Lys 352, Arg 307 Pi-pi interaction: Trp 314
Synapsin III	Ursolic acid	−5.73	H Bonds: Gly 312, Asn 313 Alkyl Bonds: Ala 316, Phe 286 Carbon-hydrogen Bond: Ser 309
Synapsin III	Donepezil	−7.28	H Bonds: Lys 106, Thr 184 Alkyl Bonds: Ala 218, Val 256, Lys 150, Phe 110, Tyr 182, Pro 107 Carbon-hydrogen bond: Gly 152, Ser 191 Pi anion: Asp 194, Lys 160

**Table 2:** MM-GBSA binding free energy of of *R. officinalis* active compounds with the target proteins.

AChE	ΔG_complex_	ΔG_receptor_	ΔG_ligand_	ΔG_bind_
Rosmarinic acid	−46,014.79	−45,849.86	−124.2334	−40.6927
Carnosic acid	−9927.076	−12,735.73	2850.116	−41.4654
Ursolic acid	−12,566.42	−12,652.49	129.8068	−43.7367
Donepezil	−12,641.44	−12,680.03	81.1693	−42.5805
**BACE**				
Rosmarinic acid	−34,885.23	−34,728.67	−132.9516	−23.6095
Carnosic acid	−5294.862	−5588.338	2835.457	−2541.98
Ursolic acid	−8016.414	−5540.046	134.2603	−2610.629
Donepezil	−8008.447	−5585.226	115.4342	−2538.656
**Synapsin-I**				
Rosmarinic acid	−28,807.57	−28,635.99	−121.7649	−49.8122
Carnosic acid	−3588.86	−6284.642	2750.101	−54.3182
Ursolic acid	−6185.364	−6295.104	132.3759	−22.6355
Donepezil	−28,678.94	−28,607.5	−48.6825	−22.7567
**Synapsin-II**				
Rosmarinic acid	−29,054.36	−28,900.69	−118.5544	−35.1146
Carnosic acid	−29,031.46	−28,950.41	−57.2692	−23.7774
Ursolic acid	−6071.435	−6187.76	134.8061	−18.4811
Donepezil	−6133.151	−6183.642	68.3527	−17.8623
**Synapsin-III**				
Rosmarinic acid	−27,429.88	−27,273.57	−123.8107	−32.4908
Carnosic acid	−2686.096	−5320.382	2641.009	−6.723
Ursolic acid	−5793.038	−5918.352	130.956	−5.6419
Donepezil	−5851.622	−5905.256	82.103	−28.4686

**Table 3:** Drug likeness of the active compounds of *Rosmarinus officinalis*.

	Carnosic Acid	Rosmarinic Acid	Ursolic Acid
**Lipinski Rule of Five**			
Molecular Mass	332.44	360.32	456.71
Hydrogen Bond Donors	1	5	2
Hydrogen Bond Acceptors	4	8	3
Log P	4.18	1.53	7.005
Molar Refractivity	98.60	81.95	152.11
**ADMET analysis**			
Human intestinal absorption	+	+	+
Caco2 permeability	+	-	+
Subcellular localization	Mitochondria	Mitochondria	Mitochondria
OATP1B1 & OATP1B3 inhibitor	+	+	+
CYP inhibitory promiscuity	-	-	-
Carcinogenicity	-	-	-
Ames mutagenesis	-	-	-
Estrogen receptor binding	+	+	+
Androgen receptor binding	-	+	+
Thyroid receptor binding	+	+	+
Glucocorticoid receptor binding	+	+	+

+ presence, - absence.

## Data Availability

All data generated or analyzed during this study are included in this published article.

## Sample Availability

Samples of the compounds are not available from the authors.
